# Mapping and QTL Analysis of Early-Maturity Traits in Tetraploid Potato (*Solanum tuberosum* L.)

**DOI:** 10.3390/ijms19103065

**Published:** 2018-10-08

**Authors:** Xingcui Li, Jianfei Xu, Shaoguang Duan, Jiaojiao Zhang, Chunsong Bian, Jun Hu, Guangcun Li, Liping Jin

**Affiliations:** Institute of Vegetables and Flowers, Chinese Academy of Agricultural Sciences/Key Laboratory of Biology and Genetic Improvement of Tuber and Root Crop, Ministry of Agriculture and Rural Affair, Beijing 100081, China; lixingcui1215@163.com (X.L.); xujianfei@caas.cn (J.X.); duanshaoguang@caas.cn (S.D.); zhangjiaojiaocaas@163.com (J.Z.); bianchunsong@caas.cn (C.B.); hujun@caas.cn (J.H.)

**Keywords:** potato, genetic segment, early maturity trait, marker development, QTL mapping

## Abstract

Early maturity is one of the most important agronomical traits in potato breeding. To identify the DNA segment that codes for early maturity, a tetraploid potato segregation population of “Zhongshu 19” × “Zhongshu 3” was genetically analyzed, using a combination of high throughput simplified genome sequencing (2b-RAD) and bulked segregant analysis (BSA). The DNA segment related to the early-maturity trait was identified at the 3.7~4.2 Mb locus on the short arm of chromosome 5. Eight molecular markers were developed, of which five were closely linked to the early-maturity trait loci. Additionally, 42 simple sequence repeats (SSR) markers were constructed based on the reference sequence of *Solanum tuberosum* group Phureja DM1-3 516 R44 (DM). Using the TetraploidMap software, the linkage map of chromosome 5 was constructed with 50 markers. The total map length was 172 centiMorgan (cM), with an average genetic distance of 3.44 cM. Correlating molecular and phenotypic data of the segregating population, the mapped Quantitative Trait Loci (QTL) on the short arm of chromosome 5 contributed to 33.55% of the early-maturity phenotype. The early-maturity QTL was located at 84 cM, flanked by the SSR5-85-1 and SCAR5-8 markers. The QTL was fine-mapped to 471 kb. Using DNA sequence annotation, 34 genes were identified in this region, 12 of them with unknown function. Among the other 22 annotated genes, E3 ubiquitin ligase gene *PUB14* could be related to maturity and regulation of tuber formation. The constructed QTL map is a useful basic tool for the cloning of early-maturity related genes in tetraploid potatoes.

## 1. Introduction

Potato (*Solanum tuberosum* L.) is the third most consumed crop worldwide and is an important industrial raw material. Maturity is one of the important characteristics for the selection of potato varieties. It is also an important agronomic trait and a major breeding target. Varieties with different maturity can meet production and consumption demand in different regions and seasons. The breeding of different maturity varieties is highly significant in the development of the potato industry. Previous studies have shown that potato maturity was controlled by minor recessive polygenes, which were distributed in 12 chromosomes [1]; because diploid potatoes have simpler genetic ratios than tetraploids, it is easy for genetic analysis and molecular operations to be carried out. Thus, the development of potato markers and genetic map construction are mainly performed at diploid level, using traditional Quantitative Trait Loci (QTL) mapping, and most of the studies mapped the major QTL for maturity in chromosome 5, linked to the QTL for resistance to late blight [2,3,4,5,6]. In 2007, a diploid potato population was used to map a QTL associated with growth stages that is closely linked to marker BA47f2t7 (P1) in chromosome 5 [1]. Later, it was found that the major QTLs that control maturity in chromosome 5 were closely related to the QTLs controlling late blight resistance, but they were independent from each other and closely linked to the GP21 marker [7]. Tetraploid potatoes (2*n* = 4× = 48) are highly heterozygous and have a high frequency of genetic recombination and a complicated genetic ratios [8]. It is much more difficult to conduct genetic analysis with tetraploid material than with diploid potatoes. In tetraploid potatoes, a major maturity-associated QTL was identified in chromosome 5, which showed a contribution rate of 54.7% to the phenotype [9]. Using the same populations, some minor QTLs with contribution rates between 5.4% and 16.5% were identified [10]. Recently, many single nucleotide polymorphisms (SNPs) markers were developed, and a high-density genetic linkage map containing 3839 SNPs was constructed by analyzing the dose information of these SNPs in tetraploid potatoes. One QTL associated with maturity, closely linked to molecular marker c2_476095, was mapped in chromosome 5 with a contribution rate of 55% [11]. Furthermore, the *StCDF1.2* gene was cloned from the short arm of chromosome 5 near maturity-associated marker GP21 [12]. *StCDF1.2* belongs to the DNA-binding One Zinc Finger transcription factor family and participates as an intermediate regulator factor in the potato tuber induction pathway.

With the development of high-throughput sequencing technologies, marker development and genetic segment mining in complex genomic species became simpler. The high-throughput simplified genome sequencing 2b-RAD technique uses DNA type II B restriction endonucleases (*Bsa*XI and *Alf*I) to cleave DNA from the upstream and downstream sites of the target site on genomic DNA and to obtain DNA fragments of consistent length (tags). The 2b-RAD technique avoids the process of fragment size selection used in other genome-wide sequencing technologies, produces more uniform tags, simplifies the development of complex genomic markers, permits mining of chromosomal segments, and helps to construct linkage and genetic variation maps in natural populations [13]. This technique has been applied to construct high-density genetic linkage maps for animals, plants, and marine organisms. For example, the high-density genetic linkage map of *Chlamys farrer* is based on 3806 molecular markers, with an average distance of 0.41 cM between markers and a genome coverage of 99.5%. Growth-and-sex-related QTLs were located on this map [14].

In this study, we used a segregating tetraploid potato population to identify genetic segments related to the early maturity trait. The segments were identified with high-throughput simplified genome sequencing (2b-RAD) and bulked segregant analysis (BSA). Molecular markers were developed for these segments and a linkage map was constructed with the TetraploidMap software [15]. For the QTL analysis, the foliage maturity data of four subsequent planting seasons (2014–2017) were used. It was concluded that the obtained linkage map will be a valuable tool for gene cloning.

## 2. Results

### 2.1. Distribution of the Maturity Trait in the Segregating Population

**Figure 1:**
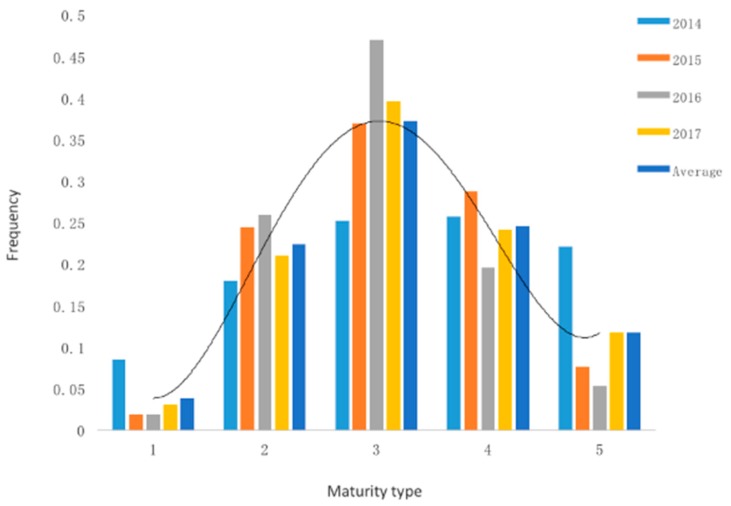
Maturity frequency distribution of the mapping population individuals. *X*-axis indicates maturity type of the mapping population, 1: Very early mature type; 2: Early mature type; 3: Middle mature type; 4: Late maturity type; 5: Very late mature type. *Y*-axis indicates the ratio between individuals of different maturity types and the whole mapping population. The entire mapping population consists of 221 individuals.

In Zhangbei (ZB), the materials were planted in early May and emerged in mid-June. Some genotypes reached their physiological maturity in late August, while others were still vigorous in late September. In Liaocheng (LC), the potatoes were planted in early March, emerged in mid-April, and some genotypes reached their physiological maturity in late June, while others were still growing and could not reach their physiological maturity due to very high temperatures. Therefore, only four-year phenotypic data collected in ZB was used for further analysis. The statistical analysis of the populations’ maturity trait (mean of four consecutive years) showed it to be consistent with the polygenic characteristics of the QTL. The offspring showed a normal distribution over the entire scoring range (Figure 1). Detailed phenotypic data are shown in Table S3. Genomic DNA was extracted from 35 very early and early maturing genotypes and 33 very late maturing genotypes, and the corresponding early maturing and late maturing DNA pool were constructed, respectively.

The significant probability of the F test indicates that there was a significant difference in maturity between 221 genotypes. Heritability in the broad sense of maturity per plot ($H^{2}/plot)$ is 0.5328 (Table 1). Heritability in the broad sense of maturity per mean ($H^{2}/mean)$ is 0.8101, indicating that the phenotypic measurement error between the years of the experiment is small. The heritability of maturity is not very high, which may be due to the fact that maturity is a quantitative trait controlled by multiple genes.

### 2.2. Screening and Marker Development for the Early Maturity Trait

Simplified genome sequencing on four samples of parents “Zhongshu 3” and “Zhongshu 19”, the early maturing pool, and the late maturing pool were carried out using the 2b-RAD technique. And a total of 52,714,218 reads were obtained. The average number of reads per sample was 13,178,554, with an average sequencing depth of 42× (Table 2). High-quality reads with *Bsa*XI digestion sites were >90% in the four sequencing libraries. An average number of 125,556 unique tags per sample were obtained.

**Figure 2:**
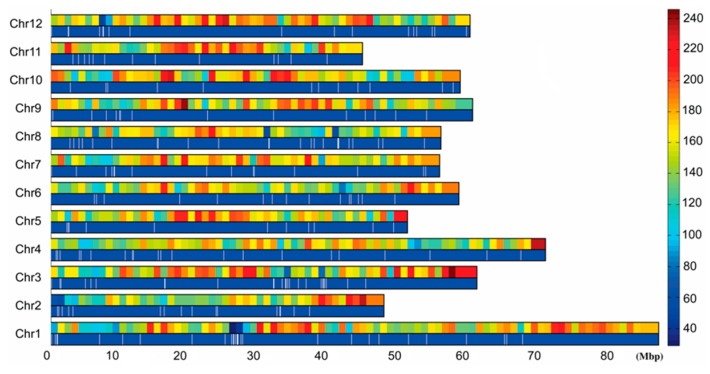
Distribution of tags on the chromosome and tag density map. 0–80 M: Chromosome physical distance (unit: M). Chr1–Chr12: 12 potato chromosomes, respectively. Right histogram: Different colors indicate different tag numbers, and numbers indicate specific tag numbers. In each chromosome, the upper long histogram shows the tag density distribution on the chromosome and the long histogram below shows the tag distribution on the chromosome.

**Figure 3:**
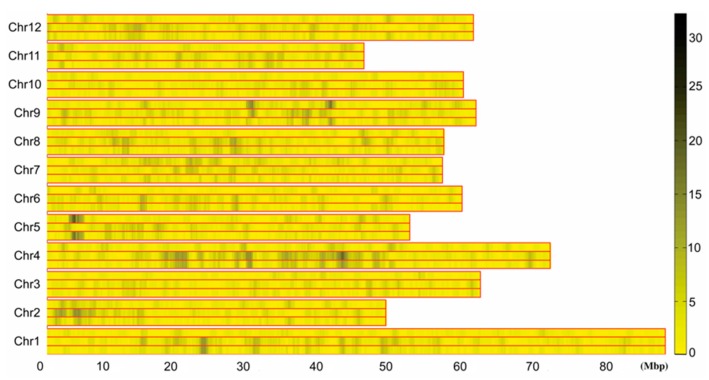
Differential tag density map of early and late maturing mixed pool. 0–80 M: Chromosome physical distance (unit: M). Chr1–Chr12: 12 potato chromosomes, respectively. In each chromosome, the upper long histogram shows the specific tag density of the early maturing pool, the middle long histogram shows the specific tag density of the late maturing pool, and the long histogram below shows the absolute value of differential tag density between the two pools. Right histogram, different colors indicate different tag numbers, and numbers indicate the specific tag numbers.

SNP marker typing was performed according to the obtained sequencing tag sequences and the tag distribution and density profiles on 12 potato chromosomes were obtained, and the results showed that the tags were evenly distributed on all 12 potato chromosomes and there was no large-scale tag information missing (Figure 2). Using the specific tag densities of the early maturing and late maturing pools, the density difference map was constructed. Segments with a large difference in tag density between early and late maturing pools were primarily located in chromosome 4 and 5. The segment with the most significant difference in tag density was located at 3.68–6.19 Mb in chromosome 5, followed by 18.6–20.9 Mb and 27.4–30.3 Mb in chromosome 4 (Figure 3). The specific tag density of the early maturing pool was relatively high in chromosome 5, suggesting that this segment may be related to the early maturity trait. The specific tag densities of the late maturing pool were relatively high in chromosome 4, which might be related to the late maturity trait (Figure 3).

Based on the specific tag position of the segments on the chromosome and the potato reference genomic sequence, a total number of 92 primers were designed, with 52 primer pairs in the 3.68–6.19 Mb region of chromosome 5, and 40 primer pairs in the differential segments of chromosome 4. Twenty polymorphic markers were developed for chromosome 5, including 4 CAPS and 4 SCAR markers. Further testing on the F1 population showed that only five markers were closely linked to the early maturity trait (SCAR5-5, SCAR5-8, CAPS5-3-2, CAPS5-21-2, and CAPS5-24). Polymorphic markers on other chromosomes were not linked to the maturity trait (data not shown), suggesting that only the genetic segment in chromosome 5 was associated with the early maturity trait. The SCAR5-8 marker in chromosome 5 was used to validate 70 early and late maturing Chinese cultivars, showing a high coincidence rate of 81.4% in marker detection results [16]. Hence, SCAR5-8 can be successfully applied in marker-assisted breeding programs of potato.

### 2.3. QTL Mapping for the Early Maturity Trait

**Figure 4:**
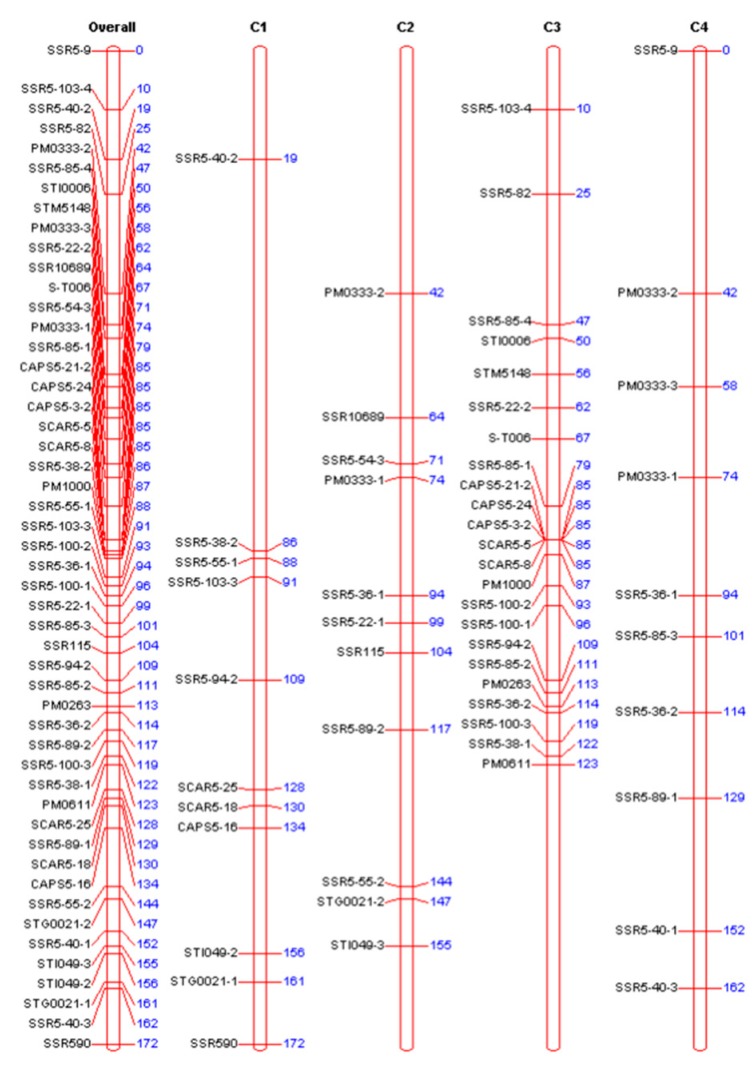
Genetic map of chromosome 5 in potato. The left side of the map is 50 molecular markers. The numbers on the right indicate the corresponding genetic distances (unit: cM). Overall: Chromosome 5. C1–C4: Represent four homologous chromosomes of chromosome 5, respectively.

To verify the accuracy of the genetic segments related to the early maturity trait, a genetic linkage map of chromosome 5 was constructed. Early and late maturing parents and their 16 progenies were used to assess the polymorphism of the 152 SSR primer pairs in chromosome 5. A total number of 32 polymorphic SSR markers were obtained. These markers and the previously developed 4 CAPS and 4 SCAR markers were used to test the F1 generation. In this study, we focused on early maturity, and a genetic linkage map for early type parent “Zhongshu 3” was constructed (Figure 4). The map included 50 markers, of which 33 were simplex, 4 were duplex, and 13 were double simplex markers (types of marker were analyzed by TetraploidMap, combined with their phenotypical segregation ratio in the offspring). The total length of the map coverage was 172 cM, with an average distance between markers of 3.44 cM. The 50 markers of this map were four CAPS markers, four SCAR markers, and 42 SSR markers, respectively. Among the 42 SSR markers, 25 were single markers, and 17 were polymorphic markers. The 25 single markers were referred as: SSR5-22-1, SSR5-22-2, SSR5-36-1, SSR5-36-2, SSR5-38-1, SSR5-38-2, SSR5-40-1, SSR5-40-2, SSR5-40-3, SSR5-55-1, SSR5-55-2, SSR5-85-1, SSR5-85-2, SSR5-85-3, SSR5-85-4, SSR5-100-1, SSR5-100-2, SSR5-103-3, SSR5-103-4, PM0333-2, PM0333-3, STI049-2, STI049-3, STG0021-1, and STG0021-2.

The QTLs for foliage maturity type were identified with the TetraploidMap software. Differences between markers were assessed individually (separated by year and trait), using the analysis of variance method (ANOVA) and the Kruskal–Wallis test [17]. A *p*-value of less than 0.01 was used as a threshold criterion for QTL detection. The results of these tests suggested the existence of QTLs for foliage mature type in chromosome 5. Additionally, 22 markers in chromosome 5 may be associated with maturity. The marker cluster CAPS 5-21-2, CAPS5-24, CAPS5-3-2, SCAR5-5, SCAR5-8 was closely linked to the QTL locus, with a *p*-value less than 0.01 and a standard error (SED) of 0.0937 (Table 3).

**Figure 5:**
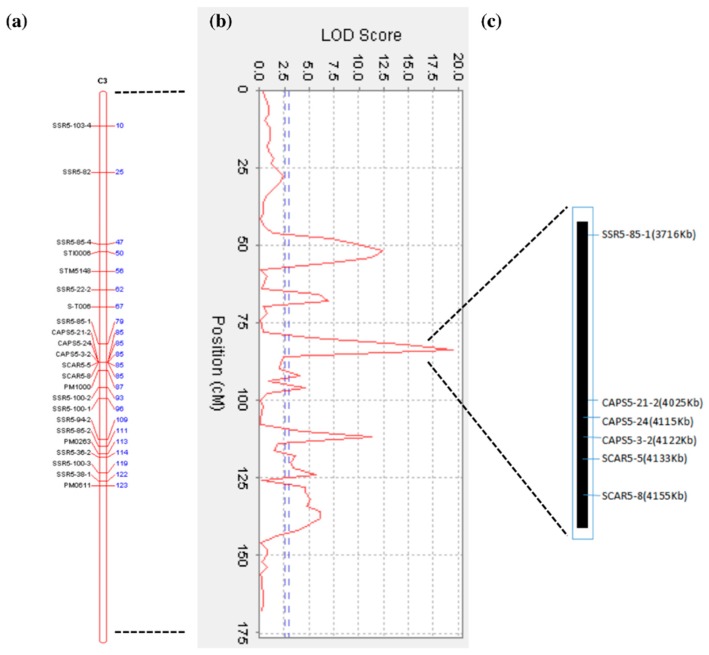
Mapping and Quantitative Trait Loci (QTL) Analysis of Early-Maturity Traits in Tetraploid Potato. (**a**) The 3rd homologous chromosome of chromosome 5; (**b**) QTL mapping for maturity trait. The horizontal line indicates the confidence threshold (LOD value = 2.97); (**c**) Physical interval of the early maturity QTL in chromosome 5 corresponding genetic map.

Using the 4 years average phenotypic data of the F1 population, three significant QTLs for early-maturity trait were identified in chromosome 5, with LOD scores above the threshold value of 2.97. The most important QTL was mapped at 84 cM of the 3rd homologous chromosome of chromosome 5, and was located between the SSR5-85-1 marker and the cluster of CAPS5-21-2, CAPS5-24, CAPS5-3-2, SCAR5-5, and SCAR5-8 (Figure 5a). The maximum LOD value of this QTL was 19.491 and explained 35.55% of the phenotypic variation. Two other loci may also be associated with maturity, one QTL linked to the STM5148 marker (LOD value of 12.50), and the other mapped at 113 cM in correspondence of PM0263 marker (LOD value of 11.30) (Figure 5b).

In summary, the QTLs for the early maturity trait were mapped on a physical interval of 471 Kb with six molecular markers, flanked by the SSR5-85-1 and the SCAR5-8 markers. The order of the markers in this QTL region is consistent with the physical order of the markers in the potato DM genome sequence. (PGSC. Tuberosum group Phureja DM1-3 Pseudomolecules (v4.03)) (Figure 5c). The identified QTL interval (471 Kb) contained 34 genes (http://solanaceae.plantbiology.msu.edu/pgsc_download.shtml), of which 22 had known and 12 had unknown functions (Table 4). The 22 annotated genes have different physiological functions and participate in different physiological regulation pathways. For example, E3 ubiquitin ligase PUB14 is involved in the photoperiodic regulatory pathway, and the auxin export carrier is involved in plant hormone transport. Heat shock protein binding, Quinolinate phosphoribosyl transferase, phosphatidylinositol kinase fyv1, resistance protein BS2, and resistance protein PSH-RGH6 are involved in plant defensive reactions. The WD repeat protein is involved in protein transport and nucleic acid processing modification. The Myb transcription factor is involved in the plant secondary metabolic regulatory pathway.

## 3. Discussion

### 3.1. Genetic Segments Mining for Early Maturity Trait, Using the 2b-RAD Sequencing Technique

Many researchers have used traditional molecular marker technologies for QTL mapping on potatoes. Most of the studies mapped major genetic loci for maturity in chromosome 5 and found that other chromosomes may also have maturity minor QTLs. In this study, for the first time, a high-throughput simplified 2b-RAD sequencing technique approach was used to identify and mark genetic segments for complex quantitative traits in potatoes. Segments with a large difference in specific tags were identified in chromosome 4 and 5, and further tag validation showed that the genetic segment in chromosome 5 is associated with the early maturity trait, which is consistent with previous studies [2,3,4,8,9,10,18].

The genetics of tetraploid potato are complex, and molecular marker development is difficult. Previously published markers were mainly developed with diploid potato [19,20,21,22]. but not directly on the tetraploid level. In this study, a total number of 52 specific primer pairs were designed and synthesized, and eight molecular markers were developed with a development efficiency of 15.4%. The eight markers were further assessed on early and late maturing progenies of the tetraploid segregation population, and five markers were found to be closely linked to the early maturity trait. It was concluded that the described method can be used for the development of markers of complex polyploid genomes, such as potato, and shorten the marker development cycle.

Although there are a few reports that mentioned the existence of a maturity-related genetic segment in chromosome 4, the tag validation results in this study showed that the genetic segment in chromosome 4 was not related to maturity. This may be due to several reasons: (a) The tetraploid potato genome is highly heterozygous and some information may not have been measured adequately (lack of sequencing depth, miss recognition of *Bsa*XI); (b) filtering out of low quality reads resulted in a partial loss of the genetic information, and eventually the differential tag interval appeared; (c) removal of tags with fewer occurrences may have caused a difference in the tag density.

### 3.2. Genetic Linkage Map Construction and QTL Mapping

A 50-marker genetic linkage map was constructed using tetraploid potato segregation population, and an early maturity trait QTL was mapped at 84 cM, near the SCAR5-8 marker (or SCAR5-5, CAPS5-3-2, CAPS5-24, CAPS5-21-2) on the short arm of chromosome 5. This means that the QTL for the early-maturity trait, obtained by genetic map mapping, is consistent with the maturity genetic segment results obtained by high-throughput simplified genome sequencing. and also proved the feasibility and accuracy of high-throughput simplified genome sequencing 2b-RAD method in mining potato genetic segments for important traits. The five molecular markers (SCAR5-8, SCAR5-5, CAPS5-3-2, CAPS5-24, CAPS5-21-25) that are closely linked to the early maturity trait loci are from the same genetic segment and are relatively close to each other. No significant separations between markers were observed in the genetic map. Furthermore, the five molecular markers were clustered on the 3rd homologous chromosome and belonged to the same linkage group. The other three molecular markers, CAPS5-16, SCAR5-18, SCAR5-25 are probably linked to the late maturity trait loci and were clustered on the 1st homologous chromosome (Figure 4).

Previous studies on mapping of maturity QTLs showed that the genetic interval related to maturity was relatively large. The major QTL for maturity was mapped at 0–6 cM in chromosome 5 in diploid potato, and the genetic distance between two flanking markers was 6 cM [23]. In tetraploid potato, the major QTL for maturity was mapped at 14–22 cM in chromosome 5, and the genetic distance between two flanking markers was 8 cM [10]. In this study, based on simplified genome genetic segment mining and marker development, the QTL for the early maturity trait was mapped in a physical interval of 471 kb. The average distance between two markers was 3.44 cM. The bioinformatical analysis revealed that there was a total number of 22 annotated genes in the 471 kb region, and the E3 ubiquitin ligase PUB14 gene was most likely related to potato maturity. In Arabidopsis, this gene regulates flowering of *Arabidopsis thaliana* [24]. Among the photoperiod, it regulates the flowering pathway. E3 ubiquitin ligase target proteins are mediated by E3 ubiquitin ligase (ubiquitination) and this may affect the photoperiod signals for flowering regulation, photoreceptor stability, circadian clock function, and flowering regulator CO stability [25,26,27]. Photoperiod has a great influence on potato plant growth, tuber formation, and development. Under long daylight, stems and leaves grow vigorously, many stolons form, but the tuber development is delayed and yield decreases. Under short photoperiod daylight, plant growth is normal, and tuber production and transport of assimilation products are faster, which results in a higher tuber yield. Early maturing varieties are sensitive to daylight, while late maturing varieties are forming their tubers under short daylight conditions. Therefore, the signal transduction pathway of potato flowering and tuber formation may be similar to those reported in the flowering regulation of *Arabidopsis thaliana.* E3 ubiquitin ligase PUB14 may act as an intermediate mediator during potato flowering or tuber formation, regulating flowering and tuber formation. The auxin export vector gene may also be involved in potato maturity. Auxins participate in the regulation and control of many physiological and biochemical processes, such as root occurrence, photoreaction, apical dominance, flowers development, leave and fruits shedding, and the distribution of assimilation products. In the process of auxin transport, this protein, as a specific auxin output vector, may induce a polar transport of indole-3-acetic acid (IAA). In the late growing and developing phase of potatoes, this gene may be involved in the control of the distribution of the assimilation product, as well as leaf and fruit shedding; thus, plants show early maturation. However, whether these two genes are related to maturity needs further analysis and validation. In this study, we also examined the segregation of the maturity-related gene *StCDF1* [11]. The late maturing parent “Zhongshu 19” contains the gene *StCDF1.1* (late maturity related gene) and *StCDF1.2* (early maturity related gene), while the early maturing parent “Zhongshu 3” only contains the *StCDF1.1*; none of parents contain *StCDF1.3* (early maturity related gene), and no polymorphism was observed in the segregating population. The *StCDF1.2* gene was tested in 83 Chinese varieties, and the results showed that only 4 out of 36 early maturity varieties and 5 out of 47 late maturity varieties contain the early maturity related gene *StCDF1.2*. The alignment of *StCDF1* with the reference genome showed that *StCDF1* is located at 4.537–4.542 Mb, but the maturity loci of the present study was at 3.7–4.2 Mb. The pedigree assessment showed that the *StCDF1.2* gene was cloned from CE3130 and came originally from the *S. phureja* species [11]. The early maturity related genes of “Zhongshu 3” were possibly coming from the cultivar “Katahdin”. The segregation population of this study probably contains other maturity-related genes, apart from the *StCDF1* family.

The tetraploid mapping software TetraploidMap used in this study can only identify three marker types [15]. For a single dominant marker, the parental genotype is AOOO × OOOO, and the segregation ratio of progeny is 1:1. For double dominant markers, the parental genotypes are AAOO × OOOO and AOOO × AOOO, and the segregation ratios are 5:1 and 3:1. There are two types of markers the software does not recognize, that is, markers of parental genotypes AOOO × AAOO and AAOO × AAOO with the segregation ratios of 11:1 and 35:1, respectively. Therefore, among the 32 polymorphic markers selected from 152 SSR markers, four markers belonged to the 11:1 marker type, and the software did not recognize them. Consequently, only 28 polymorphic SSR markers were used for the construction of the map. Nevertheless, the QTL for the early maturity trait was mapped in the physical region of 471 kb, and the genes contained in this region were analyzed adequately. The present study provides a solid base for future cloning initiative of major genes related to early maturity in potatoes.

## 4. Materials and Methods

### 4.1. Plant Materials

The mapping population contained 221 individuals, which were obtained from a cross between two tetraploid varieties: “Zhongshu 19” × ”Zhongshu 3”. The female parent “Zhongshu 19” is a late-maturing variety with a growth period of 110 days after emergence. The pedigree of “Zhongshu 19” is (CIP92.187 × CIP93.154) × (Bierma × Colmo). The male parent “Zhongshu 3” is an early-maturing variety with a growth period of 75 days and pedigree of (Jingfeng 1 × BF77A). Both varieties are common cultivated varieties released from our breeding program and widely grown in China.

### 4.2. Field Trials

The field trials were carried out in Zhangbei (ZB), Hebei Province (41°15’ N, 114°07’ E, 1500 m a.s.l., China), and Liaocheng (LC), Shandong Province (36°45’ N, 115°97’ E, 38 m a.s.l., China) during four consecutive planting seasons (2014–2017). The average minimum and maximum temperatures in ZB and LC ranged from 10.05 °C to 21.66 °C and 12.59 °C to 24.37 °C, respectively. The day length duration was 8.54 h in ZB and 6.45 h in LC. A completely randomized design was adopted in our study. In early May (ZB) and mid-March (LC), four tubers were planted for each of the progenies. The tubers were harvested in late September (ZB) and late June (LC).

### 4.3. Phenotypic Identification

Growth period (GP) was defined as the time span between seedling emergence and physiological maturation (50% of the plant leaves show yellow coloration). Emergence of individual seedlings was assessed every five days, starting 20 days after planting. Physiological maturity of the plants was assessed every five days, starting 60 days after emergence (DAE). The plants of the F1 population were classified by its growth period on a scale from one to five, 1: Very early mature (GP of less than 70 days); 2: Early mature (GP of 71–80 days); 3: Medium-early mature (GP of 81–100 days); 4: Late mature (GP of 101–110 days); 5: Very late mature (GP of more than 110 days).

### 4.4. DNA Extraction and Construction of Mixed Progeny Pools

Samples of young leaves were collected from “Zhongshu 3”, “Zhongshu 19”, and the F1 generation. The genomic DNA was extracted using the cetyl trimethyl ammonium bromide (CTAB) method. DNA quality was assessed with 1% agarose on a BioDrop UV/VIS spectrophotometer (SERVA, Heidelberg, Germany). For the construction of the mixed progeny pool, tissue was cut out of young leaves using a punch (Ø: 10 mm). For each F1 generation, one single leaf was used, which was collected from the same location of each plant. The tissues of very early and early maturity clones (<75 days) were mixed in equal proportions, and the DNA of the mixed tissues was extracted for simplified genome sequencing. The DNA mix of very late mature clones (>110 days) was prepared, as well as that of very early and early maturity clones. DNA extraction and quality assessment were performed the same as the above.

### 4.5. Simplified Genome Sequencing Analysis and Marker Development

A high-throughput simplified genomic 2b-RAD sequencing technique was used to construct a tag sequencing library of the parental DNA and the progeny DNA pool. Single-terminal sequencing was performed on the HiSeq 2500 V2 platform (Illumina, San Diego, CA, USA). Sequences without *Bsa*XI recognition sites, of low quality or with more than ten consecutive identical bases, were removed from the original reads. The individual high quality reads were mapped to the DM1-3 516 R44 (hereafter referred to as DM) potato reference genome using the Short Oligonucleotide Analysis Package (SOAP). Number and depth of specific tags that can be used for genotyping were identified, and genome-wide SNP screening and genotyping was performed. Based on the classification results, a chromosome tag density distribution map of four samples was constructed, using MATLAB software; further, we constructed specific tag density distribution and absolute value distribution of the specific tag density between the two mixed pools of very early and early maturity DNA pool and very late maturity DNA pool, respectively, and then screened out chromosome segments with a large difference in tag densities. Based on the tag information of the different segments, synthetic primers were designed according to the genomic sequences published on the potato genome sequence website (http://solanaceae.plantbiology.msu.edu/cgi-bin/gbrowse/potato/). The quality and specificity of the primers were assessed using the genomic DNA of the late maturing parent “Zhongshu 19” and early maturing parent “Zhongshu 3” as templates. The amplified products were detected by agarose gel electrophoresis with a concentration of 1.2%. The PCR reaction system consisted of: 5.6 μL ddH_2_O, 1.0 μL buffer (10× PCR), 0.8 μL dNTPs (10 mmol·L^−1^), 0.2 μL forward primer (10 μmol·L^−1^), 0.2 μL reverse primer (10 μmol·L^−1^), 0.2 μL Taq enzyme (2.5 U·μL^−1^), and 2 μL DNA (25 ng·μL^−1^). PCR was performed with the following cycle: 94 °C for 3 min, followed by 35 cycles of 94 °C for 30 s, 59 °C for 30 s, and 72 °C for 50 s, and finally, 72 °C for 10 min.

The PCR products were digested by *Bsa*XI restriction endonuclease if the primers amplified clear and nondifferent bands in the parents (“Zhongshu 3” and “Zhongshu 19”). The enzyme digestion system was composed of 8 μL PCR product, 1.5 μL CutSmart^®^ buffer (New England Biolabs, MA, USA), 0.2 μL *Bsa*XI endonuclease (0.5 μL/U), and 5.3 μL ddH_2_O. The primers whose PCR products can be digested only in one parent and not digested in the other parent were used to develop cleaved amplified polymorphic sequence (CAPs) markers. The primers that amplified a band in one parent but not in the other parent, or amplified different size bands in the two parents, were used to develop sequence characterized amplified region (SCAR) markers. The progeny of early and late maturing clones was used for validation.

### 4.6. Genetic Map Construction and QTL Mapping

The developed molecular markers here, as well as a set of 152 previously published SSR markers (in chromosome 5) were screened for polymorphism, based on the genomic DNA templates of “Zhongshu 3”, “Zhongshu 19”, and 16 F1 individuals [28,29,30]. The selected polymorphic markers were assessed and validated with a population of 221 progenies. Three types of markers were selected for map construction with the TetraploidMap software [15]: (1) Simplex dominant markers (segregating 1:1) with a *p* value less than 0.01 for the Chi-square test; (2) duplex dominant markers (segregating 5:1), with a *p* value higher than 0.01; (3) double-simplex dominant markers (segregating 3:1) with a *p* value higher than 0.01, and which were linked to at least one simplex marker. Markers with a segregation ratio of 11:1 (simplex × duplex) were omitted from the linkage map. Markers were ordered by proper sequences with the TetraploidMap software [17]. Fifty selected markers were assigned to the four homologous chromosomes, considering the coupling or repulsion relationship between markers [17]. The primer sequence data of the markers are listed in Table S1, and genotype information for 221 progenies is listed in Table S2.

## Figures and Tables

**Table 1:** Variance analysis of phenotypic data.

Source of Variation	DF	SS	MS	F	*p*-Value	$H^{2}/plot\;$	$H^{2}/mean\;$
Genotype	220	584.0126	2.6546	5.2672	3.53 × 10^−59^	0.5328	0.8101
Error	606	305.47	0.5040				
Total	826	889.4293					

DF: Degree of freedom; SS: Sum of square; MS: Mean square; F: F-value; $H^{2}/plot$: Heritability in the broad sense of maturity for per plot; $H^{2}/mean$: Heritability in the broad sense of maturity for per mean.

**Table 2:** Basic analysis of sequencing data.

Material	Original Reads	High Quality Reads	Number of Unique Tags	Depth of Unique Tags
Zhongshu 3	12,698,244	11,764,321	121,769	92.60%
Zhongshu 19	12,411,142	11,829,691	119,455	95.30%
Early maturity pool	13,849,678	12,952,153	127,237	93.50%
Late maturity pool	13,755,154	12,425,805	128,556	90.30%

**Table 3:** Markers associated with physiological maturity.

Marker	Segregation Ratio	Dosage	KWSig	AVSig	Mean(0)	Mean(1)	SED
SCAR5-8	1:1	S	0.0000	0.0000	3.61	2.65	0.0937
SCAR5-5	1:1	S	0.0000	0.0000	3.61	2.65	0.0937
CAPS5-3-2	1:1	S	0.0000	0.0000	3.61	2.65	0.0937
CAPS5-24	1:1	S	0.0000	0.0000	3.61	2.65	0.0937
CAPS5-21-2	1:1	S	0.0000	0.0000	3.61	2.65	0.0937
SCAR5-18	1:1	S	0.0000	0.0000	2.91	3.45	0.1087
SCAR5-25	1:1	S	0.0000	0.0000	2.94	3.46	0.1087
CAPS5-16	1:1	S	0.0001	0.0000	2.98	3.46	0.1112
S-T006	1:1	S	0.0000	0.0000	3.46	2.79	0.1062
SSR590	1:1	S	0.0045	0.0029	3.02	3.36	0.1131
STG0021-1	1:1	S	0.0016	0.0010	2.99	3.36	0.1127
STI0006	1:1	S	0.0000	0.0000	3.47	2.74	0.1057
STI049-2	1:1	S	0.0028	0.0019	3.01	3.38	0.1162
PM0611	1:1	S	0.0000	0.0000	3.63	2.90	0.1114
PM1000	3:1	DS	0.0000	0.0000	3.71	2.91	0.1182
PM0263	1:1	S	0.0000	0.0000	3.53	2.82	0.1037
STM5148	1:1	S	0.0000	0.0000	3.49	2.85	0.1114
SSR5-55-1	3:1	DS	0.0016	0.0026	2.84	3.27	0.1387
SSR5-85-2	3:1	DS	0.0000	0.0001	3.62	3.05	0.1437
SSR5-100-1	3:1	DS	0.0000	0.0000	3.62	3.01	0.1278
SSR5-100-2	3:1	DS	0.0000	0.0000	3.70	3.00	0.1304
SSR5-100-3	1:1	S	0.0000	0.0000	2.88	3.48	0.1103

DS: double simplex marker; S: simplex marker; KWSig: The significance of Kruskal–Wallis test. AVSig: The significance of the analysis of variance. Mean: The mean of maturity score. Mean (0): The mean when the marker is absent. Mean (1): The mean when the marker is present. SED: The standard error of difference between the means.

**Table 4:** Gene annotation and location in the physical interval.

Start Position	Terminal Position	Gene ID	Gene Function
3794799	3797561	PGSC0003DMG400030514	Histone chaperone ASF1A
3800502	3803746	PGSC0003DMG400030561	HB06p
3804570	3804947	PGSC0003DMG400042076	Gene of unknown function
3808243	3812259	PGSC0003DMG400030560	ATP synthase subunit beta
3812966	3817781	PGSC0003DMG400030559	Fruit protein PKIWI502
3819129	3825291	PGSC0003DMG400030513	Sarcoplasmic reticulum histidine-rich calcium-binding protein
3826477	3835460	PGSC0003DMG400030558	ALG2-interacting protein X
3846875	3847435	PGSC0003DMG400035916	Conserved gene of unknown function
3855942	3861025	PGSC0003DMG40003051	1Repressor of RNA polymerase III transcription MAF1
3872716	3876866	PGSC0003DMG400030557	Membrane associated ring finger 1,8
3897559	3898635	PGSC0003DMG400030556	Gene of unknown function
3903471	3909624	PGSC0003DMG400030510	Conserved gene of unknown function
3927363	3930287	PGSC0003DMG400030509	Ribose-5-phosphate isomerase
3943033	3943669	PGSC0003DMG400030508	By genscan and genefinder
3945081	3955925	PGSC0003DMG400030507	Abc transporter
3960120	3964565	PGSC0003DMG400030555	Acetylglucosaminyl transferase
3966729	3968667	PGSC0003DMG400030554	Pentatricopeptide repeat protein
3969058	3970107	PGSC0003DMG400030506	FLA20
3975677	3980357	PGSC0003DMG400030553	ATP binding
3991348	3993998	PGSC0003DMG400030552	Chloroplast-targeted copper chaperone
4009033	4012803	PGSC0003DMG400030551	Spore coat protein
4016212	4017445	PGSC0003DMG400030505	Conserved gene of unknown function
4025454	4033006	PGSC0003DMG400030550	Conserved gene of unknown function
4033954	4042260	PGSC0003DMG400030549	Conserved gene of unknown function
4046634	4053740	PGSC0003DMG400030548	Myb-like transcription factor 6
4055474	4059978	PGSC0003DMG400030547	E3 ubiquitin ligase PUB14
4062234	4063922	PGSC0003DMG400030504	Conserved gene of unknown function
4068242	4070295	PGSC0003DMG400030503	Conserved gene of unknown function
4083569	4086820	PGSC0003DMG400030546	Conserved gene of unknown function
4088794	4097482	PGSC0003DMG400030502	Fyve finger-containing phosphoinositide kinase, fyv1
4097590	4098620	PGSC0003DMG400030582	Gene of unknown function
4107529	4113266	PGSC0003DMG400030544	WD-repeat protein
4111951	4118400	PGSC0003DMG400030501	Quinolinate phosphoribosyl transferase
4169120	4173221	PGSC0003DMG400030543	Gene of unknown function

## Supplementary Materials

Supplementary materials can be found at http://www.mdpi.com/1422-0067/19/10/3065/s1.
