# Supplementary material for: Mapping and QTL Analysis of Early-Maturity Traits in Tetraploid Potato (Solanum tuberosum L.)
Source: Int J Mol Sci. 2018 Oct 8;19(10):3065. doi: 10.3390/ijms19103065 (PMC6213731; doi:10.3390/ijms19103065)
Supplement: Supplementary file 1 [file ijms-19-03065-s001.zip › Supplementary Files/Table S1 .docx]

**Table S1** Information of the 50 primers used in the genetic map

| Markers | Primer sequence 5'-3' | Tm℃ | Target length (bp) | Restriction enzyme |
| --- | --- | --- | --- | --- |
| SCAR5-8 | CACTTCGTAAAAATCCTGG | 59 | 581 |  |
|  | ATTCATCCCATCACGTTTT |  |  |  |
| SCAR5-5 | GTATATGGACGTATCAAAA | 59 | 842 |  |
|  | TGATAACATTAGTTGGGTA |  |  |  |
| SACR5-18 | CATTAAGGAGGCTGATTAAAGC | 59 | 912 |  |
|  | CACTTTGCAGCACGACTACCAA |  |  |  |
| SCAR5-25 | TGTATCAGCTTCTTCAAGGTTT | 59 | 868 |  |
|  | CTATACTTCATCCATGGCTGTT |  |  |  |
| CAPS5-3-2 | ACTCCACTATGCAAGTCCTATT | 56 | 944 | BsaXI |
|  | CCTTGTGTTAATGCATATGTGC |  |  |  |
| CAPS5-16 | ACATCCTACCAAACGACCCTTC | 59 | 820 | BsaXI |
|  | ACTGTCCTGATTTGAAGTCCTT |  |  |  |
| CAPS5-21-2 | CCTTTTAATTTCTCTTTGTGAT | 56 | 582 | BsaXI |
|  | ATGGTGATATGAGGTTTATGAA |  |  |  |
| CAPS5-24 | GGAATATACCAGGTGTCCAATT | 56 | 709 | BsaXI |
|  | CTTTTTGAGGAAACATACACCC |  |  |  |
| SSR5-9 | AGCACAAAACTCACACTTCCA | 58 | 143 |  |
|  | GGCCTACAAAGGGGGCAA |  |  |  |
| SSR5-22-1 | TCCATCAATCCCCTTCTCCCT | 60 | 200 |  |
|  | GAAACTGTTTTACTTGCCCCC |  |  |  |
| SSR5-22-2 | TCCATCAATCCCCTTCTCCCT | 60 | 190 |  |
|  | GAAACTGTTTTACTTGCCCCC |  |  |  |
| SSR5-36-1 | TGGACTTTCGGACACTTGTGA | 56 | 132 |  |
|  | CCTTGCATTTCGAGAGGGC |  |  |  |
| SSR5-36-2 | TGGACTTTCGGACACTTGTGA | 56 | 123 |  |
|  | CCTTGCATTTCGAGAGGGC |  |  |  |
| SSR5-38-1 | TGCGGGTTGGTGTTGTGT | 58 | 185 |  |
|  | CCGGAGGCAGTTTTTGGGA |  |  |  |
| SSR5-38-2 | TGCGGGTTGGTGTTGTGT | 58 | 203 |  |
|  | CCGGAGGCAGTTTTTGGGA |  |  |  |
| SSR5-40-1 | TCTCCTTCAAACACCGCGA | 58 | 145 |  |
|  | TCGGACACCCATTTGCCC |  |  |  |
| SSR5-40-2 | TCTCCTTCAAACACCGCGA | 58 | 175 |  |
|  | TCGGACACCCATTTGCCC |  |  |  |
| SSR5-40-3 | TCTCCTTCAAACACCGCGA | 58 | 203 |  |
|  | TCGGACACCCATTTGCCC |  |  |  |
| SSR5-54-3 | TCGCGTGAGTTTCAACACT | 56 | 206 |  |
|  | AGCGAATTTCAGAAACCTCCT |  |  |  |
| SSR5-55-1 | CACCTTGGGGAGCAGCTC | 59 | 164 |  |
|  | GCGGTGGGTTGATTTCAGG |  |  |  |
| SSR5-55-2 | CACCTTGGGGAGCAGCTC | 59 | 145 |  |
|  | GCGGTGGGTTGATTTCAGG |  |  |  |
| SSR5-82 | GCGCTTAGCCCCTTGAGT | 60 | 97 |  |
|  | CCCCAGACCCCACCTAGT |  |  |  |
| SSR5-85-1 | TGAGCCTGCATGCATTCCT | 58 | 249 |  |
|  | ACCCCATGTGTCCCGAGA |  |  |  |
| SSR5-85-2 | TGAGCCTGCATGCATTCCT | 58 | 260 |  |
|  | ACCCCATGTGTCCCGAGA |  |  |  |
| SSR5-85-3 | TGAGCCTGCATGCATTCCT | 58 | 274 |  |
|  | ACCCCATGTGTCCCGAGA |  |  |  |
| SSR5-85-4 | TGAGCCTGCATGCATTCCT | 58 | 291 |  |
|  | ACCCCATGTGTCCCGAGA |  |  |  |
| SSR5-89-1 | ACGCGGTCTCTACATCGAG | 59 | 225 |  |
|  | TGGGATATCCTGCAGTGGC |  |  |  |
| SSR5-89-2 | ACGCGGTCTCTACATCGAG | 59 | 233 |  |
|  | TGGGATATCCTGCAGTGGC |  |  |  |
| SSR5-94 | TGGTTAGGGAAAACAGCTCTT | 56 | 143 |  |
|  | TGGGGTTGCTTTTACCTGGT |  |  |  |
| SSR5-100-1 | TCCACGCAAACAATCGTTTCA | 56 | 195 |  |
|  | ACGATTAGCCCAAATCCACTT |  |  |  |
| SSR5-100-2 | TCCACGCAAACAATCGTTTCA | 56 | 204 |  |
|  | ACGATTAGCCCAAATCCACTT |  |  |  |
| SSR5-100-3 | TCCACGCAAACAATCGTTTCA | 56 | 245 |  |
|  | ACGATTAGCCCAAATCCACTT |  |  |  |
| SSR5-103-3 | GGGGTCTTCGTCGGCAAT | 58 | 162 |  |
|  | ACTGCAAGCACATGGGCT |  |  |  |
| SSR5-103-4 | GGGGTCTTCGTCGGCAAT | 58 | 191 |  |
|  | ACTGCAAGCACATGGGCT |  |  |  |
| STM5148 | TCTTCTTGATGACAGCTTCG | 55 | 414 |  |
|  | ACCTCAGATAGTTGCCATGTCA |  |  |  |
| STI0006 | CTTTAGTCCTTGGCAGAGCTT | 57 | 210 |  |
|  | CGGGCTGATTCTTCTTCATC |  |  |  |
| STI049-2 | GGAAGTCCTCAACTGGCTG | 58 | 137 |  |
|  | TCAACTATATGCCTACTGCCCAA |  |  |  |
| STI049-3 | GGAAGTCCTCAACTGGCTG | 58 | 157 |  |
|  | TCAACTATATGCCTACTGCCCAA |  |  |  |
| S_T006 | CTTTAGTCCTTGGCAGAGCTT | 57 | 199-250 |  |
|  | CGGGCTGATTCTTCTTCATC |  |  |  |
| SSR115 | CACCCTTTATTCAGATTCCTCT | 57 | 211-223 |  |
|  | ATTGAGGGTATGCAACAGCC |  |  |  |
| SSR590 | CAAATCGCGACATGTGTAAGA | 56 | 161 |  |
|  | GGAAGAGAAACGCGGACATA |  |  |  |
| SSR10689 | CGCAAAATCCAGACAAAGTT | 58 | 547 |  |
|  | GAAGGAAATGTTGGAGGAAGTG |  |  |  |
| PM1000 | GGAATCGATCAAAGCAAATCAGTT | 60 | 193 |  |
|  | CAATGTGGAATTTGTTTCCTCACA |  |  |  |
| PM0611 | GCATTGAGCACTAAAGGAGGCTAA | 60 | 158 |  |
|  | TCGCAGTAGTTACCTGCATTTCTTT |  |  |  |
| PM0263 | TCGCAGTAGTTACCTGCATTTCTTT | 60 | 133 |  |
|  | TCATTTGAAGTACATTTACACCATGTTT |  |  |  |
| PM0333-1 | GGGTGGGGTATTTAAAACACGATA | 55 | 183 |  |
|  | TGGTCCTAACTCCACAACACTTCA |  |  |  |
| PM0333-2 | GGGTGGGGTATTTAAAACACGATA | 55 | 197 |  |
|  | TGGTCCTAACTCCACAACACTTCA |  |  |  |
| PM0333-3 | GGGTGGGGTATTTAAAACACGATA | 55 | 220 |  |
|  | TGGTCCTAACTCCACAACACTTCA |  |  |  |
| STG0021-1 | TGCCTACTGCCCAAAACATT | 56 | 136 |  |
|  | ACTGGCTGGGAAGCATACAC |  |  |  |
| STG0021-2 | TGCCTACTGCCCAAAACATT | 56 | 125 |  |
|  | ACTGGCTGGGAAGCATACAC |  |  |  |
