# Supplementary material for: Mapping and QTL Analysis of Early-Maturity Traits in Tetraploid Potato (Solanum tuberosum L.)
Source: Int J Mol Sci. 2018 Oct 8;19(10):3065. doi: 10.3390/ijms19103065 (PMC6213731; doi:10.3390/ijms19103065)
Supplement: Supplementary file 1 [file ijms-19-03065-s001.zip › Supplementary Files/Table S3 .docx]

**Table S2** Phenotypic data 2014-2017

| _Year_  _NO.individuals_ | 2014 | 2015 | 2016 | 2017 | Mean |
| --- | --- | --- | --- | --- | --- |
| 1 | 2 | 2 | 2 | 2 | 2.00 |
| 2 | 5 | 3 | 5 | 5 | 4.50 |
| 3 | 3 | 3 | 2 | 2 | 2.50 |
| 4 | 5 | 5 | 4 | 4 | 4.50 |
| 5 | 5 | 4 | 3 | 4 | 4.00 |
| 6 | 4 | 4 | 3 | 5 | 4.00 |
| 7 | 3 | 3 | 3 | 3 | 3.00 |
| 8 | 2 | 3 | 3 | 4 | 3.00 |
| 9 | 2 | 2 | 2 | 3 | 2.25 |
| 10 | 5 | 4 | 5 | 5 | 4.75 |
| 11 | 1 | 4 | 5 | 4 | 3.50 |
| 12 | 3 | 3 | 3 | 3 | 3.00 |
| 13 | 3 | 5 | 3 | 4 | 3.75 |
| 14 | 5 | 4 | 4 | 4 | 4.25 |
| 15 | 3 | 3 | 4 | 4 | 3.50 |
| 16 | 5 | 4 | 4 | -99 | 4.33 |
| 17 | 3 | 4 | 3 | 3 | 3.25 |
| 18 | 4 | 4 | 3 | 3 | 3.50 |
| 19 | 3 | 4 | -99 | -99 | 3.50 |
| 20 | 1 | 3 | 3 | 3 | 2.50 |
| 21 | 3 | 2 | 2 | 3 | 2.50 |
| 22 | 2 | 3 | 2 | 3 | 2.50 |
| 23 | 4 | 3 | 5 | 5 | 4.25 |
| 24 | 4 | 3 | 3 | 3 | 3.25 |
| 25 | 4 | 3 | 3 | 3 | 3.25 |
| 26 | 4 | 5 | 4 | 5 | 4.50 |
| 27 | 4 | 4 | 3 | 4 | 3.75 |
| 28 | 3 | 3 | 3 | 3 | 3.00 |
| 30 | 5 | 4 | 4 | 4 | 4.25 |
| 31 | 5 | 4 | 4 | 4 | 4.25 |
| 32 | 3 | 2 | 2 | 2 | 2.25 |
| 33 | 3 | 2 | 4 | 3 | 3.00 |
| 34 | 5 | 4 | 3 | 4 | 4.00 |
| 35 | 4 | 4 | 3 | 4 | 3.75 |
| 36 | 4 | 3 | 3 | 3 | 3.25 |
| 37 | 5 | 4 | 5 | 4 | 4.50 |
| 38 | 4 | 5 | 3 | 3 | 3.75 |
| 39 | 4 | 4 | 3 | 4 | 3.75 |
| 40 | 2 | 3 | 3 | 4 | 3.00 |
| 41 | 4 | 5 | 3 | 3 | 3.75 |
| 42 | 2 | 2 | 3 | 3 | 2.50 |
| 43 | 1 | 2 | -99 | -99 | 1.50 |
| 44 | 5 | 5 | 4 | 5 | 4.75 |
| 45 | 3 | -99 | 3 | 3 | 3.00 |
| 46 | 4 | 3 | 3 | 4 | 3.50 |
| 47 | 4 | 4 | 4 | 4 | 4.00 |
| 48 | 3 | 3 | 4 | 3 | 3.25 |
| 49 | 1 | 1 | 2 | 2 | 1.50 |
| 50 | 3 | 3 | 3 | 3 | 3.00 |
| 51 | 5 | 4 | 3 | 5 | 4.25 |
| 52 | 5 | 4 | 4 | 4 | 4.25 |
| 53 | 3 | 4 | 4 | 3 | 3.50 |
| 54 | 5 | 4 | 5 | 5 | 4.75 |
| 55 | 2 | -99 | 1 | -99 | 1.50 |
| 56 | 1 | 2 | 2 | 2 | 1.75 |
| 57 | 4 | 3 | 3 | 3 | 3.25 |
| 58 | 4 | 5 | 2 | 4 | 3.75 |
| 59 | 1 | 2 | 2 | 2 | 1.75 |
| 60 | 5 | 4 | 4 | 5 | 4.50 |
| 61 | 2 | 1 | 1 | 2 | 1.50 |
| 62 | 2 | 2 | 3 | 3 | 2.50 |
| 63 | 5 | 3 | 3 | 3 | 3.50 |
| 64 | 2 | 3 | 3 | -99 | 3.00 |
| 65 | 2 | 2 | 3 | 3 | 2.50 |
| 66 | 4 | 4 | 3 | 3 | 3.50 |
| 67 | 1 | -99 | -99 | -99 | 1.00 |
| 69 | 5 | 4 | 3 | -99 | 4.00 |
| 70 | 3 | 5 | 2 | 3 | 3.25 |
| 71 | 1 | 2 | 1 | 2 | 1.50 |
| 72 | 2 | 2 | 4 | 3 | 2.75 |
| 73 | 2 | 3 | 5 | 1 | 2.75 |
| 74 | 5 | 3 | 3 | 3 | 3.50 |
| 75 | 4 | 4 | 2 | 3 | 3.25 |
| 76 | 5 | 4 | 3 | 5 | 4.25 |
| 77 | 5 | 3 | 3 | 4 | 3.75 |
| 78 | 4 | 2 | 2 | 5 | 3.25 |
| 81 | 5 | 3 | 2 | 5 | 3.75 |
| 82 | 1 | 3 | 3 | 3 | 2.50 |
| 83 | 4 | 2 | 3 | 3 | 3.00 |
| 84 | 3 | 2 | 3 | 3 | 2.75 |
| 85 | 1 | 2 | 3 | 2 | 2.00 |
| 86 | 4 | 2 | 2 | 3 | 2.75 |
| 87 | 3 | 3 | 3 | 3 | 3.00 |
| 88 | 5 | 3 | 3 | 3 | 3.50 |
| 89 | 3 | 4 | 3 | 3 | 3.25 |
| 90 | 3 | 3 | -99 | -99 | 3.00 |
| 91 | 1 | 3 | 3 | 3 | 2.50 |
| 92 | 4 | 2 | 4 | 4 | 3.50 |
| 93 | 3 | 2 | 2 | 4 | 2.75 |
| 94 | 5 | 4 | 4 | 5 | 4.50 |
| 95 | 4 | 4 | 3 | 2 | 3.25 |
| 96 | 3 | 3 | 3 | 2 | 2.75 |
| 97 | 5 | 3 | -99 | -99 | 4.00 |
| 98 | 3 | 4 | -99 | -99 | 3.50 |
| 99 | 4 | 3 | 3 | 3 | 3.25 |
| 100 | 4 | 4 | 2 | 4 | 3.50 |
| 101 | 2 | 3 | 2 | 2 | 2.25 |
| 102 | 4 | 4 | -99 | -99 | 4.00 |
| 103 | 2 | 3 | 2 | 2 | 2.25 |
| 104 | 3 | 2 | -99 | -99 | 3.00 |
| 105 | 2 | 2 | 2 | 2 | 2.00 |
| 106 | 2 | 3 | 3 | 2 | 2.50 |
| 107 | 1 | 3 | 1 | -99 | 2.00 |
| 108 | 5 | 2 | -99 | -99 | 3.50 |
| 109 | 1 | 3 | 2 | 2 | 2.00 |
| 110 | 3 | 2 | 3 | 3 | 2.75 |
| 111 | 5 | 2 | 5 | 5 | 4.25 |
| 112 | 3 | 4 | 3 | 2 | 3.00 |
| 113 | 5 | 4 | 3 | 3 | 3.75 |
| 114 | 4 | -99 | -99 | -99 | 4.00 |
| 115 | 4 | -99 | -99 | -99 | 4.00 |
| 116 | 3 | 3 | 3 | 3 | 3.00 |
| 117 | 2 | 3 | 2 | 2 | 2.25 |
| 118 | 5 | 3 | 3 | 5 | 4.00 |
| 119 | 5 | 4 | 3 | 5 | 4.25 |
| 120 | 5 | 3 | 3 | 3 | 3.50 |
| 121 | 1 | 3 | 2 | 1 | 1.75 |
| 122 | 2 | 4 | 2 | 2 | 2.50 |
| 123 | 1 | 3 | 3 | 3 | 2.50 |
| 124 | 2 | 2 | 2 | -99 | 2.33 |
| 125 | 5 | 4 | 4 | 4 | 4.25 |
| 126 | 5 | 4 | 4 | 4 | 4.25 |
| 127 | 4 | 4 | 4 | 4 | 4.00 |
| 128 | 5 | -99 | 4 | 4 | 3.33 |
| 129 | 4 | 5 | 4 | 3 | 4.00 |
| 130 | 5 | 3 | 4 | 4 | 4.00 |
| 131 | 4 | 3 | 2 | 3 | 3.00 |
| 132 | 3 | 2 | 2 | 2 | 2.25 |
| 133 | 2 | 1 | 2 | 1 | 1.50 |
| 134 | 2 | 2 | 2 | 2 | 2.00 |
| 135 | 3 | 3 | 3 | 3 | 3.00 |
| 136 | 2 | 2 | 2 | 2 | 2.00 |
| 137 | 2 | 3 | 2 | 3 | 2.50 |
| 138 | 4 | 5 | 3 | 3 | 3.75 |
| 139 | 2 | 2 | 2 | 3 | 2.25 |
| 140 | 3 | 3 | 3 | 3 | 3.00 |
| 141 | 4 | 4 | 3 | 3 | 3.50 |
| 142 | 4 | 4 | 4 | 4 | 4.00 |
| 143 | 4 | 2 | 3 | 3 | 3.00 |
| 144 | 5 | 5 | 4 | 4 | 4.50 |
| 145 | 2 | -99 | 2 | 2 | 2.00 |
| 146 | 5 | -99 | -99 | -99 | 5.00 |
| 147 | 1 | -99 | -99 | -99 | 1.00 |
| 148 | 4 | 2 | -99 | -99 | 3.00 |
| 149 | 5 | 4 | 4 | 4 | 4.25 |
| 150 | 5 | 4 | 5 | 5 | 4.75 |
| 151 | 4 | 3 | 3 | 4 | 3.50 |
| 153 | 2 | 2 | 2 | 2 | 2.00 |
| 154 | 4 | 3 | 4 | 3 | 3.50 |
| 155 | 1 | 1 | 2 | 2 | 1.50 |
| 156 | 5 | 5 | 4 | 4 | 4.50 |
| 157 | 2 | 3 | 2 | 3 | 2.50 |
| 158 | 3 | 4 | 3 | 4 | 3.50 |
| 159 | 4 | 2 | 3 | 2 | 2.75 |
| 160 | 4 | 4 | 3 | 4 | 3.75 |
| 161 | 2 | 2 | 2 | -99 | 2.00 |
| 162 | 3 | 3 | 3 | 2 | 2.75 |
| 163 | 3 | 3 | 3 | 3 | 3.00 |
| 164 | 3 | 3 | 2 | 2 | 2.50 |
| 165 | 3 | 3 | 3 | 3 | 3.00 |
| 166 | 2 | 2 | 2 | 2 | 2.00 |
| 167 | 2 | 2 | 2 | 2 | 2.00 |
| 168 | 2 | -99 | 2 | 2 | 2.00 |
| 169 | 3 | 4 | 3 | 4 | 3.50 |
| 170 | 5 | 5 | 5 | 5 | 5.00 |
| 171 | 4 | 4 | 3 | 3 | 3.50 |
| 172 | 4 | 4 | 4 | 4 | 4.00 |
| 173 | 4 | 5 | 2 | -99 | 3.67 |
| 174 | 4 | 4 | 3 | 4 | 3.75 |
| 175 | 2 | -99 | 2 | 2 | 2.00 |
| 176 | 3 | 3 | 3 | 3 | 3.00 |
| 177 | 5 | 4 | -99 | -99 | 4.50 |
| 178 | 2 | 3 | 2 | 3 | 2.50 |
| 179 | 4 | 3 | 4 | 5 | 4.00 |
| 180 | 5 | 5 | 4 | 4 | 4.50 |
| 181 | 3 | -99 | -99 | -99 | 3.00 |
| 182 | 5 | 3 | 3 | 3 | 3.50 |
| 183 | 3 | 3 | 3 | 3 | 3.00 |
| 184 | 3 | 2 | 2 | 3 | 2.50 |
| 186 | 4 | 4 | 3 | 3 | 3.50 |
| 187 | 5 | 4 | 4 | 4 | 4.25 |
| 188 | 2 | 3 | 2 | 1 | 2.00 |
| 189 | 3 | 2 | 2 | 1 | 2.00 |
| 190 | 4 | 3 | 3 | -99 | 3.33 |
| 191 | 4 | 3 | 3 | 3 | 3.25 |
| 192 | 1 | 2 | 2 | 4 | 2.25 |
| 193 | 3 | 3 | 3 | 3 | 3.00 |
| 194 | 3 | 2 | 3 | 3 | 2.75 |
| 195 | 2 | 2 | 3 | 3 | 2.50 |
| 196 | 4 | 4 | 3 | 4 | 3.75 |
| 197 | 4 | 3 | 3 | 4 | 3.50 |
| 198 | 5 | 4 | 4 | 4 | 4.25 |
| 199 | 3 | 3 | 3 | 3 | 3.00 |
| 200 | 2 | 2 | 3 | 2 | 2.25 |
| 201 | 3 | 2 | 3 | 2 | 2.50 |
| 202 | 3 | 3 | 4 | 3 | 3.25 |
| 204 | 2 | 2 | 2 | 2 | 2.00 |
| 205 | 4 | 2 | 2 | 1 | 2.25 |
| 206 | 3 | 3 | 3 | 3 | 3.00 |
| 207 | 3 | 3 | 3 | 3 | 3.00 |
| 208 | 5 | 4 | 5 | 5 | 4.75 |
| 209 | 4 | 2 | 3 | 3 | 3.00 |
| 210 | 2 | 2 | 3 | 3 | 2.50 |
| 212 | 3 | 3 | 3 | 3 | 3.00 |
| 213 | 5 | 2 | 4 | 5 | 4.00 |
| 214 | 3 | -99 | -99 | -99 | 3.00 |
| 215 | 4 | 4 | 4 | 3 | 3.75 |
| 216 | 3 | 3 | 3 | 3 | 3.00 |
| 217 | 3 | 3 | 3 | 2 | 2.75 |
| 218 | 1 | 2 | 2 | 2 | 1.75 |
| 219 | 4 | 3 | 3 | -99 | 3.33 |
| 220 | 3 | 3 | 3 | 4 | 3.25 |
| 221 | 4 | 4 | 4 | 4 | 4.00 |
| 222 | 3 | 4 | 4 | 3 | 3.50 |
| 223 | 5 | 3 | 2 | 2 | 3.00 |
| 224 | 2 | 2 | 2 | 2 | 2.00 |
| 225 | 3 | 4 | 3 | 2 | 3.00 |
| 226 | 3 | 3 | 3 | 2 | 2.75 |
| 227 | 4 | 4 | 4 | 4 | 4.00 |
| 228 | 5 | 5 | 3 | 5 | 4.50 |
| 230 | 4 | 3 | 4 | 5 | 4.00 |
| Notice：missing value be coded as -99 | | | | | |
